# Primary mitochondrial disease in the US: Data from patients and physicians' perspective on health care delivery

**DOI:** 10.1016/j.dib.2019.104343

**Published:** 2019-07-30

**Authors:** Amel Karaa, Amy Goldstein, Cristy Balcells, Kira Mann, Laura Stanley, Philip E. Yeske, Sumit Parikh

**Affiliations:** a Department of Genetics, Massachusetts General Hospital, Boston, MA, USA; b Human Genetics, Children's Hospital of Philadelphia, Philadelphia, PA, USA; c MitoAction, Boston, MA, USA; d Foundation for Mitochondrial Medicine, Atlanta, GA, USA; e United Mitochondrial Disease Foundation, Pittsburgh, PA, USA; f Neurogenetics, Center for Pediatric Neurology, Cleveland Clinic Children's Hospital, Cleveland, OH, USA

**Keywords:** Mitochondrial disease, Health care delivery, Patients' perception of care, Physicians' opinion on care delivery

## Abstract

This article presents data that examine the patient's perception of health care delivery for mitochondrial disease in the US. It also presents the opinions of mitochondrial disease expert physicians about creating a specialised network of clinics to oversee the care of patients with this disease within the US. Two separate electronic surveys were developed; one for mitochondrial disease patients and their families ascertaining their satisfaction with their current health care and the challenges they face. The other for the physicians group assessing the usefulness, feasibility and readiness to develop specialized care clinics for mitochondrial disease in the US. Survey responses and descriptive analysis are presented here. The data in this article is supplemental, and supports the information presented in the research article “Harmonizing care for rare diseases: How we developed the mitochondrial care network in the United States.” Karaa et al., 2019

**Table:** Specifications Table

Subject	Health
Specific subject area	Health care delivery for rare disease.
Type of data	Table
How data were acquired	Electronic survey
Data format	Raw, analysed with descriptive statistics
Parameters for data collection	All data obtained from the surveys of patients and physicians was analysed.
Description of data collection	Data was collected electronically using an email link.
Data source location	Country: Patients and physicians residing in the US
Data accessibility	With the article
Related research article	Author's name: Amel Karaa, Amy Goldstein, Cristy Balcells, Kira Mann, Laura Stanley, Philip E. Yeske, Sumit Parikh Title: Harmonizing care for rare diseases: How we developed the mitochondrial care network in the United States. Journal: Molecular genetics and metabolism https://doi.org/10.1016/j.ymgme.2019.05.012

**Table:** 

**Value of the Data** • This data provides a first look at the patients and physicians' perception of care delivery for primary mitochondrial disease in the US and the challenges they face. • Health care providers and practice managers will find this data very useful in better understanding the health care gaps within their institutions. • These data are the first step to identify the challenges and the gaps in health care delivery for mitochondrial disease in the US and will serve as road map to improve those challenges and fill those gaps [1].

## 1 Data

The data set presented includes survey responses from 1) patients with self-reported mitochondrial disease (MD) and their caregivers and 2) US physicians, these are physicians expert in mitochondrial diseases, practicing within the United States. Table 1 shows the 14 multiple choice and open-ended questions (left column) of the patients' survey assessing MD disease duration, relationship of patients and their families with their health care providers, communication within the health care team and limitations of care delivery. Subjects responses are summarized in the middle column of Table 1 with the number of responses (N) and the percentage of the responses to each specific question presented on the right, last 2 columns. The open-ended responses to the last question are presented in Table 2.

**Table 1:** Patients' perspective on their mitochondrial health delivery by their care team.

Questions		N/243	%
How long ago were you or your family member first diagnosed with mitochondrial disease?	18 months or less	33	13.6
	2–5 years	83	34.1
	Over 5 years	127	52.3
Which of the following best represents the most significant limitation for your relationship with your primary care provider (PCP)/pediatrician? (choose one)	I do not have enough time to discuss all my issues with my PCP/pediatrician during the visit.	33	13.6
	I do not think my PCP/pediatrician understands mitochondrial disease well enough to treat me.	131	54
	My PCP/pediatrician is not always available to take my calls or see me.	21	8.6
	My PCP/pediatrician does not feel comfortable dealing with my health concerns because I/my child am/is too complex	39	16
	My PCP is a pediatrician and says I/my child am/is too old for the practice, but I can't find a new adult PCP to agree to take me on.	8	3.3
	I do not feel I can trust my PCP/pediatrician	5	2
	I do not have a PCP.	6	2.5
How often do you see your primary care provider (PCP)/pediatrician?	Every 3 months	68	28
	Every 6 months	48	19.7
	Once a year	44	18
	Only when necessary due to illness	83	34.3
How many specialists do you or your child/family member see for your healthcare needs?	One	16	6.6
	Two	23	9.5
	Three or more	203	84
Do you or your child/family member have a mitochondrial disease specialist?	Yes	151	62
	No	58	24
	I/my child/family member had a mitochondrial disease specialist in the past but do not have one now	34	14
If you have a mitochondrial disease specialist, which of the following responses best represents the limitation of your relationship with him/her?	My specialist is too busy; he/she can't see me frequently or acutely if I get sick suddenly.	42	17.3
	My specialist is located far away from where I live; I can't drive/fly to see him/her as often as I would like to	64	26.3
	My specialist is not involved in my care when I get admitted to a hospital where he/she is not affiliated to.	8	3.2
	My specialist does not communicate with my PCP/pediatrician and other providers.	24	10
	My PCP/pediatrician and/or other providers don't communicate with my Mito specialist.	35	14.4
	I do not have a mitochondrial disease specialist.	70	28.8
Which healthcare provider do you feel is in charge of you or your child/family member's healthcare needs?	Primary care provider (PCP)/pediatrician	80	33
	Another specialist	44	18
	Mitochondrial specialist	51	21
	No one	68	28
Are you satisfied that your/family member's healthcare is well organized and that your healthcare providers work together?	Yes	79	32.5
	No	164	67.5
How often were you given confusing or contradictory information about your/family member's healthcare treatments?	Never	12	5
	Rarely	48	19.7
	Sometimes	103	42.3
	Frequently	80	33
What, if any, negative impacts have the limitations with your healthcare provider that you listed on your/your child's health? (Please choose up to 3 answers)	Mental health (anxiety, depression, anger)	80	33
	Ability to walk/move/participate in daily activities	64	26.3
	Sleep	33	13.6
	Ability to eat/digest food normally	57	23.5
	Energy level	111	45.7
	Pain level	62	25.5
	Interactions with other people, including classmates, teachers, community and family	17	7
	Disease progression	91	37.5
	I do not feel there has been any negative health impact	39	16
If your primary care provider (PCP)/pediatrician and mitochondrial disease specialist could communicate more efficiently together and collaborate more actively to treat you, what would be the key changes that you would find most helpful? (Please chose up to 3 answers).	My PCP/pediatrician and Mito specialist would directly talk to each other every time one of them sees me so that they could go over the plan with each other directly.	110	45.3
	My PCP/pediatrician and Mito specialist would ideally see me at the same time so that a common plan can be made at the time of the visit.	40	16.5
	My PCP/pediatrician and Mito specialist would both be available when I become acutely sick so that they can manage me together.	94	38.7
	My PCP/pediatrician and Mito specialist would both be involved when I get admitted to the hospital so that they can be actively involved in my care.	94	38.7
	I don't want to bother my Mito specialist for every problem. I think my PCP/pediatrician should be comfortable enough to help me, but I would like the option that my PCP/pediatrician can consult with my Mito specialist if he/she has questions/concerns	110	45.3
	I would like to see my Mito specialist more often.	45	18.5
If you were offered a well coordinated medical team involving your primary care provider (PCP)/pediatrician and mitochondrial disease specialist who would work together to address your medical needs more efficiently, what would be the TWO best measures of improved quality of life for you/your child that you would perceive as most meaningful? In other words, what type of improvement would be most meaningful to you as a direct result of this improved coordinated medical care? (Please chose up to 3 answers).	Mental health (anxiety, depression, anger)	83	34.2
	Ability to walk/move/participate in daily activities	73	30
	Sleep	22	9
	Ability to eat/digest food normally	44	18.1
	Energy level	141	58
	Pain level	69	28.4
	Interactions with other people, including classmates, teachers, community and family	33	13.6

**Table 2:** Selected open-ended responses from the patients' perspective survey.

Negative outcome from lack of communication and joint decision making between doctors taking care of MD patient (open ended question)
Unnecessary appointments with doctors who did not understand MD.
My MD specialist is a researcher and rarely gets involved in the management of mitochondrial disease.
Lack of continuity of care by seeing different doctors in a practice/academic center
Doctors on my team can't agree on the cause of my symptoms and no one wants to take responsibility or take ownership of my management.
Have had multiple instances of meds being prescribed by one specialist to manage specific symptoms, only to have another type of specialist tell us the med is detrimental to my son's health.
My PCP/local doctors refuse to follow the orders of my out of state MD doctor
We have more knowledge about MD than our doctors. Our frustration is that we manage the care between each of our specialists. This has been a great stress, never knowing if we are doing all that we should be for our son and what impact that is having on his progression.
It is very difficult to coordinate care by myself - I am a single parent and am trying to hold a full-time job in addition to spending hours coordinating care for my daughter.
My PCP doesn't know anything about MD so she just does what he says to do. It would be great if all PCP were trained in MD.
Yes, my child has been prescribed treatments by the MD specialist that the PCP did not know about or understand. The PCP has also been forced to care for my child in ways she feels are “over her head.” As a result, the PCP cannot defend care decisions for my child when other doctors ignorant of my child's needs criticize or challenge them.
It is like being in the middle of the ocean alone … a very hopeless feeling.
It makes me feel very alone. I am forced to treat myself and hope for the best.
It has delayed care. Lack of coordinated care has resulted in it taking years to receive treatment where it should have taken weeks.
I, have been in the middle of two specialists battling over a health decision for my child on more than one occasion. It leads me to not trust one of my child's doctors, to feel I have to choose sides. It leads to much added worry and stress!
Managing my child's health is a full-time job. Walking on eggshells with unsympathetic/disinterested medical professionals while my child is in pain.
Often, I hear that we are too complex, and they try to get someone else to take on the care. However, there isn't really someone else to go to.
It has created lot of anxiety during critical times.
I see a different specialist every time I have an appointment, I have to explain my condition to them over and over as they don't know what it is, its symptoms, or how it should be treated.
As a patient, it's like being a diplomat negotiating a peace treaty between my doctors.

Table 3 represents responses to the 11 questions (left column) asked of US physicians about the importance of establishing a specific MD health care network [1]. The answers from the 44 responding physicians are presented in the middle column with the number of respondents (N) and the percentage of each specific response to each question (right columns).

**Table 3:** Physicians' perspective on the development of a mitochondrial care expert network.

A2	Responses	N/44	%
Do you think that centers of excellence for mitochondrial disease should be created in the US?	Yes	41	93.2
	No	3	6.8
Do you think that a center of excellence needs to offer:	Clinical services only	12	27.3
	Both clinical and research service	32	72.7
Do you think a center of excellence needs to service both children and adults?	Just children	4	9.1
	Just adults	0	0
	Both clinical and research service	40	90.9
Does a center of excellence need to provide diagnostic services and comprehensive initial evaluation for newly diagnosed patients?	Yes	42	95.5
	No	2	4.5
Does a center of excellence need to provide follow up and management of patients?	Yes	40	93
	No	3	7
Does a center of excellence need to provide Inpatient care/support as needed	Yes	41	93.2
	No	3	6.8
What services do you think need to be offered in a center of excellence? (Check all that apply)	Coordinated care within the institution with access to subspecialists	44	100
	Coordinated care with outside providers	38	86.4
	Arrangements for transitional care from children's to adults' services	37	84.1
	Emergency Access (on call service)	35	79.5
	Education (for patients, community, other providers)	40	90.9
	Well defined standard of care Protocols for emergency visits, anesthesia …	41	93.2
	On Site Lab with access to biochemical and genetic testing	20	45.5
	Access to patients registries	37	84.1
	Access to clinical trials on site	32	72.7
	Access to state of the art genetic testing (as covered by the patient's insurance)	39	88.6
Please list any other services you think need to be offered at a center of excellence.	Local patient support groups		
	Connection to palliative care services		
	Access to social work services for families		
	Database for all centers to keep track of diagnoses and patients nationally.		
	Access to clinical trials on site		
	Multidisciplinary input (nurse, therapists, social worker, dietician)		
What personnel should be available in a center of excellence (Check all that apply)?	Geneticist	42	95.5
	Neurologist	43	97.7
	Genetic counselor	38	86.4
	Clinical coordinator	39	88.6
	Social worker	36	81.8
	Nutritionist	35	79.5
	Therapists (OT, PT, Speech)	30	68.2
	Insurance coordinator	25	56.8
List any other key personnel	Social worker for school advocacy, insurance concerns/disability		
	Family practice NP, internist, or pediatrician		
	On site ED		
	Inborn error of metabolism specialists (pediatricians/internal medicine) for both children and adults		
	Advanced practice nurses		
	Dedicated perioperative team		
	Exercise testing/kinesiology,		
Please list the essential core of specialists that need to be available in a center of excellence? (List all)	Ophthalmology/Neuro-ophthalmology, Audiology, Pulmonology with specialty in hypersalivation/aspiration.		
	Orthopedics with specialty in spasticity and scoliosis		
	Movement Disorder clinic with expertise in ataxia, chorea/dystonia and spasticity		
	Neuroradiology, Anesthesia, GI/Motility team, Feeding team, Nutrition, Endocrinology, psychiatry, maternal-fetal medicine, urology, intensivists.		
	Autonomic specialist (covered either by neurologist or cardiologist), developmental specialist		
	Biostatistical and clinical trial expertise		
	Infectious disease, Rheumatology, Neuromuscular		
What kind of well-defined standard of care protocols should be available through a center of excellence? (Check all that apply)	Emergency visits	43	97.7
	Anesthesia	42	95.5
	Surgery	33	75
	Sick protocol	42	95.5
Please list all other protocols you think need to be available in a center of excellence	Surgery should include peri-operative management with a dedicated team that will manage fluids/fasting status with expertise in metabolism.		
	Postpartum care, vaccines.		
	Protocols should be vetted by experts from around country and published if possible		
	Consistent diagnostic and treatment criteria used across centers; consistent labeling of patients if diagnosis is not genetically confirmed, consistent preventative/maintenance care		
	School related materials, seizure management suggestions and drugs to avoid		
	Dehydration, dysautonomia exacerbation.		
	Acute stroke		
	Supplement use		
	Anesthesia		
	Long distance travel		
	Biomarker & Nutritional monitoring		
	MELAS stroke-like episode		
Do you have any other suggestions?	Child Advocacy team for complicated social issues		
	I think this is a bad idea. This issue will drain our energy.		
	Periodic webinars or conferences to teach providers who will be seeing patients on a local level		
	Rigid requirements for centers will insure that there are very few centers.		
	A certain number of exceptions is needed. In particular, specific allowances should be made, such as children's hospitals are permitted to have cut off ages of 21 or older.		
	There is little incentive for institutions to provide the missing requirements since Mitochondrial Medicine loses money, so I doubt that MMS set standards will result in an increase in the quality of care provided by any institution.		
	Perversely, standards may further limit access.		
	Finding ways to make the field economically viable is the only way to improve care, and even to keep what little we have. Good Mito care is expensive. However, society pays for ICU care and neurosurgery, which are even more expensive. Theoretically, what we provide should also by recognized as valuable and appropriately reimbursed. Until we find a way to get here, care for MD patients will remain severely restricted.		

OT: Occupational therapy, PT: Physical therapy, ED: emergency department, MD: mitochondrial disorders, NP: nurse practitioner, MELAS: mitochondrial encephalomyopathy with lactic acidosis and stroke-like episodes, MMS: mitochondrial medicine society, ICU: intensive care unit, GI: gastro-enterologist.

## 2 Experimental design, materials, and methods

Information from 2 electronic survey instruments were obtained from 1) patients with mitochondrial disease (MD) and their caregivers and 2) US physicians, experts in mitochondrial diseases.

1) Patients' survey

### 2.1 Survey design

The survey instrument was intended for patients with MD and their family members or caregivers. A total of 14 multiple-choice and open-ended questions were developed to capture MD duration since diagnosis, relationship of patients and their families with their health care providers, communication within the health care team and limitations of care delivery. The questions were inspired by several discussions with patients and families and hearing about their concerns conveyed through MD advocacy groups representatives who interact with these patients continuously and from treating physicians managing these patients in clinic.

### 2.2 Participants and recruitment

The survey was administered electronically through MitoAction; a MD patient advocacy group, email list server. These are self-reported MD subjects who agreed to receive news and study notifications from MitoAction. The survey was sent to 360 subjects and responses were obtained from 243 subjects (67.5% response rate) with complete answers (Supplemental Table 1).

### 2.3 Statistical analysis

Descriptive statistics were used. All raw data was obtained from self-response queries entered by participants.

1) Physicians' survey

### 2.4 Survey design

After review of the patients' survey responses, evaluation amongst the Mitochondrial Medicine Society (MMS) board members and informal discussions with several national mitochondrial disease colleagues across the US led to the creation of a second survey. The MMS is a non-profit, physician-led organization which develops medical and clinical guidelines for the diagnosis and treatment of MD. The physician survey contained 11 multiple-choice and open-ended questions to assess the interest of US MD physicians in establishing MD care centers. The survey specifically asked about how they thought such a center would function within their own specific health care system and how differing their “wish list” for such a center would be when compared to that of the patients and their caregivers (Supplemental Table 2).

### 2.5 Participants and recruitment

The survey was sent by email to the MMS membership of more than 200 physicians and requested that only US physicians participate. Answers from 44 respondents was received.

### 2.6 Statistical analysis

Descriptive statistics were used. All raw data was obtained from self-response queries entered by participants.
